# Global climate change driven by soot at the K-Pg boundary as the cause of the mass extinction

**DOI:** 10.1038/srep28427

**Published:** 2016-07-14

**Authors:** Kunio Kaiho, Naga Oshima, Kouji Adachi, Yukimasa Adachi, Takuya Mizukami, Megumu Fujibayashi, Ryosuke Saito

**Affiliations:** 1 Department of Earth Science, Tohoku University, Sendai, Japan; 2 Meteorological Research Institute, Tsukuba, Japan; 3 Ecological Engineering Laboratory, Tohoku University, Sendai, Japan

## Abstract

The mass extinction of life 66 million years ago at the Cretaceous/Paleogene boundary, marked by the extinctions of dinosaurs and shallow marine organisms, is important because it led to the macroevolution of mammals and appearance of humans. The current hypothesis for the extinction is that an asteroid impact in present-day Mexico formed condensed aerosols in the stratosphere, which caused the cessation of photosynthesis and global near-freezing conditions. Here, we show that the stratospheric aerosols did not induce darkness that resulted in milder cooling than previously thought. We propose a new hypothesis that latitude-dependent climate changes caused by massive stratospheric soot explain the known mortality and survival on land and in oceans at the Cretaceous/Paleogene boundary. The stratospheric soot was ejected from the oil-rich area by the asteroid impact and was spread globally. The soot aerosols caused sufficiently colder climates at mid–high latitudes and drought with milder cooling at low latitudes on land, in addition to causing limited cessation of photosynthesis in global oceans within a few months to two years after the impact, followed by surface-water cooling in global oceans in a few years. The rapid climate change induced terrestrial extinctions followed by marine extinctions over several years.

The Chicxulub impact crater in the Yucatan Peninsula, Mexico, is the third largest known impact crater (ca. 180 km in diameter) on Earth. The crater was formed by the impact of an asteroid (ca. 10 km in diameter) at the Cretaceous/Paleogene (K/Pg) boundary 66 million years ago, which is marked by a layer of enhanced levels of iridium (Ir)^1^,^2^,^3^,^4^. The extinction of the dinosaurs probably occurred at the K/Pg boundary based on the statistics of their biostratigraphic distribution^5^. Only 12% of the land-dwelling forms survived, but 90% of species in the freshwater assemblage survived, because land-dwelling forms were dependent on primary productivity while the fresh water assemblage relied on detritus^6^. In the oceans, the extinction of the ammonites probably occurred just above the K/Pg boundary^7^,^8^. Marine plankton diversity and marine productivity decreased significantly, accompanied by a biogeochemical collapse, but 90% of benthic foraminiferal species survived the impact, because while there were significant environmental changes in the surface waters, there was little change in the deep sea^9^,^10^,^11^,^12^,^13^,^14^.

The globally intense infrared radiation from ballistically reentering ejecta may have killed unsheltered organisms directly and ignited fires in locations where adequate fuel was available^15^. However, the self-shielding from thermal radiation by an impact-ejecta curtain may have prevented widespread wildfire ignition^16^. The current hypothesis for their extinction is that an asteroid impact formed condensed sulfuric acid aerosols in the stratosphere, which reflected sunlight, causing global darkness and inducing cessation of photosynthesis, global near-freezing conditions (impact winter), and acid rain^1^,^17^,^18^,^19^. However, if this had occurred, crocodilians and various other animals would have also gone extinct. Recent impact experiments and model calculations have demonstrated that condensed sulfuric acid aerosols cannot form and persist over long periods following asteroid impacts^20^. The process leading to the extinction of the dinosaurs and various marine groups such as the ammonites and most planktonic foraminifera, accompanied by the survival of mammals, birds, crocodilians, and other species, is therefore unknown.

Soot is a strong light-absorbing aerosol that can have a significant climatic influence^21^,^22^, and the atmospheric lifetime of soot particles is longer (on the scale of years) in the stratosphere, although they are efficiently removed from the atmosphere by precipitation (about a week) in the troposphere^22^,^23^. However, the amount of soot in the stratosphere, the coincidence of the extinction and stratospheric soot, and the climatic influence are unknown. To clarify these points, we examined organic molecules and their isotopes in a proximal K/Pg section in Haiti accompanied by coarse-ejecta beds containing microspherules overlain by marlstone, including an iridium (Ir) accumulation layer^24^ (Supplementary Information and Supplemental Fig. 1), and a distal site without coarse-ejecta beds in Spain^25^ and then performed climate model calculations. Soot contains polyaromatic hydrocarbons (PAHs), such as coronene^26^, which form from the incomplete combustion of hydrocarbons. We estimated the total amount of soot ejected by the impact using the combusted five- to six-ring PAHs [coronene (Cor), benzo(e)pyrene (BeP), benzo(g,h,i)perylene (Bpery)] that remained in the stratosphere. There are two possible origins for the soot at the K/Pg boundary: wildfires^21^,^27^ and impact target rock^28^,^29^. The amount of the stratospheric soot estimated here includes the soot from both wild fires and impact target rock. We postulate that all of the stratospheric soot was sourced from the target rock in Chicxulub, Mexico in the model calculation, because (1) most soot particles from present-day wildfire emissions are injected in the troposphere but not into the stratosphere^30^, and (2) the sedimentary organic molecule composition indicates the same source for the PAHs from the troposphere and stratosphere, suggesting an enormous cloud of smoke from the Chicxulub crater (see “Origin of soot” in Discussion).

## Results

### Combusted PAHs

**Figure 1:**
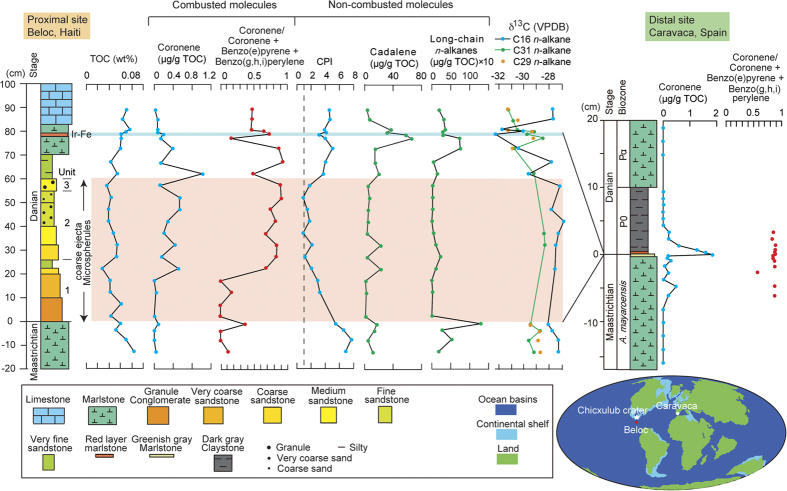
Stratigraphic variation in organic molecules derived from combustion and land plants, the Carbon Preference Index (CPI), and stable carbon isotope ratios. Cadalene and long-chain *n*-alkanes are according to Mizukami *et al*.^25^. The map shows the paleolocations of the impact site and the sections evaluated. The base map is according to Courtillot *et al*.^67^.

Coronene, a six-ring PAH, is a hydrocarbon-derived geochemical diagnostic for combustion. Coronene levels (μg/g TOC) were high in the coarse deposits and the Ir layer relative to other strata, and peaked in the fine-grained sediments in the sandstone and Ir layers (Fig. 1). The presence of both coronene and glass spherules in the coarse deposits implies that the impact induced extensive combustion. The coronene content in combusted five- to six-ring PAHs is defined by the coronene/(coronene + benzo(e)pyrene + benzo(g,h,i)perylene) [Cor/(Cor + BeP + Bpery)] ratio. The Cor/(Cor + BeP + Bpery)] ratio is extremely high in the K/Pg ejecta, that is, the upper 40 cm of the coarse-ejecta sediments (average: 0.69; Fig. 1), the Ir layer at Beloc, Haiti (0.73), and the Ir layer (0.88) and in the vicinity of the Ir layer (−2 to + 4 cm) at Caravaca, Spain (0.87 average; Fig. 1). By contrast, low coronene content (0.2–0.4) was found in the Maastrichtian deposits at Beloc. The high Cor/(Cor + BeP + Bpery) values at the K/Pg boundary are likely due to an impact with the formation of coronene as the final product, with low values more typical of wildfires^31^. The similar values of Cor/(Cor + BeP + Bpery) in the coarse-ejecta sediments, Ir layer, and at Caravaca imply the same source of coronene for the coarse-ejecta sediments and the Ir layer for the proximal and distal sites.

### Carbon preference index

The carbon preference index (CPI)^32^,^33^,^34^ values of *n*-alkanes were used to distinguish between crude oil and dead organisms. The CPI of mature *n*-alkanes is 1 (oil is mature)^32^,^33^, and the CPI of immature *n*-alkanes derived from plants is high^32^. The CPI values from the ejecta beds at Beloc decreased from 7 in the first unit to 1 to 2 in the second and third units, increased to 5 in the lower part of the Danian marlstone, decreased to a minimum of 3 in the Ir layer, and then increased to 5 in the Danian limestone (Fig. 1). These values show that the primary source of *n*-alkanes in the second and third units of the coarse ejecta deposits was crude oil, and that the *n*-alkanes in the Ir layer were sourced from both crude oil and dead organisms. The low CPI values suggest that the organisms from which the oil was derived were older than the plants at the time of the impact. This is consistent with >80% of the recovered Cantarell oil being derived from the Late Jurassic^35^.

### Carbon isotope ratios of *n*-alkanes and a land plant biomarker

The δ^13^C Vienna Pee Dee Belemnite (VPDB) standard of C16 short *n*-alkanes in Haiti decreased significantly (by 4‰) in the marlstone, reaching a minimum in the Ir layer (Fig. 1 and Supplemental Table 1), which implies that a significant devastation of marine organisms occurred after the deposition of coarse ejecta and ended with the deposition of the Ir layer.

The δ^13^C VPDB values for C29 and C31 long-chain *n*-alkanes decreased (by 3‰) from just above the coarse deposits to just above the Ir layer, with high values in the Ir layer and its vicinity (Fig. 1). The high values are considered to be derived from reworked Cretaceous plants, because the δ^13^C VPDB values were the same as those in the Maastrichtian deposits. The rebound of δ^13^C values coincided with a high coronene ratio (Fig. 1), implying that the devastation of land vegetation occurred during the marine extinction period at low latitudes. The land plant biomarker cadalene is concentrated within and just below the Ir layer, with levels four-fold higher than those in the Maastrichtian deposits, also implying that land vegetation was devastated (Fig. 1)^25^. The peak in cadalene was also recognized at the K-Pg boundary in Caravaca, Spain^36^, implying semiglobal devastation of land plants.

### Amount of soot in the stratosphere

Because the proportion of soot ejected into the troposphere and stratosphere by the impact is unknown^37^, the amount of soot ejected into the stratosphere has not been determined previously. We estimated the amount of soot in the stratosphere based on the amount of soluble Cor + BeP + Bpery, which are components of soot^38^, in fine sediments containing Ir at a proximal site. We estimated 500, 1500, and 2600 Tg black carbon (BC) as the total soot ejecta (Supplementary Information). These estimates did not include rapid removal processes (within several days) in the stratosphere, probably due to coagulation between BC and other larger-sized materials (e.g., dust), and therefore, the actual BC emission from the impact could be greater. However, because we estimated the mass of the BC ejecta from soluble five- to six-ring PAHs falling from the stratosphere during the few years after the impact, the amount of stratospheric BC, which could have caused climate change over several years, did not change. The amount of burned carbon at Chicxulub was estimated to be 1800–6000 Tg^39^, which may have decreased due to removal associated with coagulation between BC and other larger-sized materials, and is consistent with our estimate of the BC amount.

### Climate change

**Figure 2:**
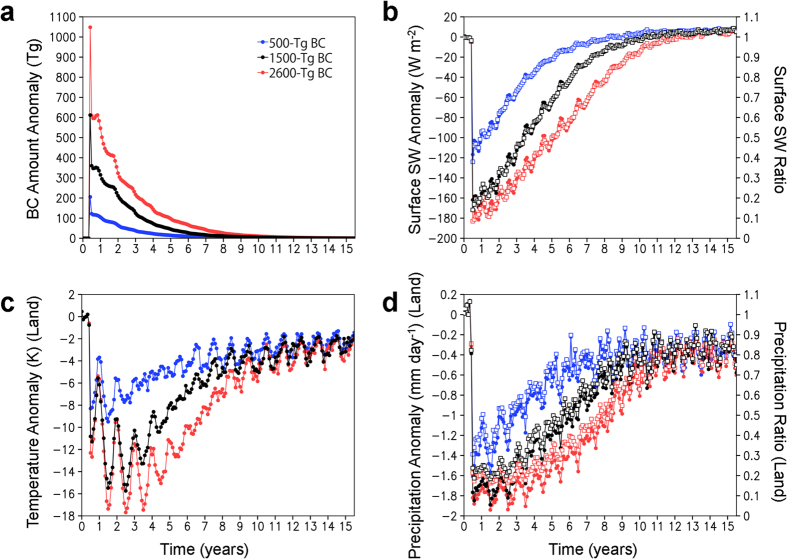
Climate changes caused by the black carbon (BC) injection. (**a–d**) Changes in the global averages of the amount of BC in the atmosphere (**a**), downward shortwave (SW) radiation at the surface (**b**), surface air temperature over the land (**c**), and precipitation over the land (**d**) for the 500-Tg (blue), 1500-Tg (black), and 2600-Tg (red) BC scenarios calculated by the climate model. Monthly anomalies from the control experiment (no ejection case) are shown on the left axis with filled circles (**a–d**), and the ratios relative to the control experiment are shown for shortwave radiation and precipitation on the right axis with open squares (**b**,**d**). The 30-year global averages of the amount of BC, downward shortwave radiation at the surface, surface air temperature over the land, and precipitation over the land in the control experiment were 41 Gg, 200 W m^−2^, 281 K, and 2.2 mm day^−1^, respectively.

**Figure 3:**
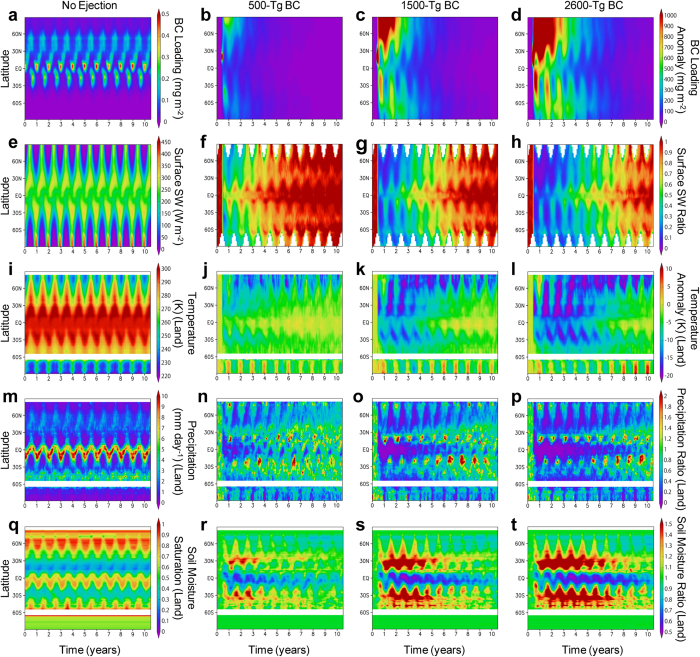
Meridional distributions of pre-industrial climate conditions and climate changes caused by the black carbon (BC) injection. (**a–t**) Time–latitude cross-sections of zonal mean changes in BC loading in the atmosphere (**a–d**), downward shortwave (SW) radiation at the surface (**e–h**), surface air temperature over land (**i–l**), precipitation over land (**m–p**), and degree of saturation of soil moisture averaged for a depth of 0–50 cm (**q–t**) for the no ejection (control) (**a**,**e**,**i**,**m**,**q**), 500-Tg (**b**,**f**,**j**,**n**,**r**), 1500-Tg (**c**,**g**,**k**,**o**,**s**), and 2600-Tg (**d**,**h**,**l**,**p**,**t**) BC scenarios calculated by the climate model. Monthly anomalies from the control experiment are shown for the amount of BC (**b–d**) and temperature (**j–l**). The ratios relative to the control experiment are shown for shortwave radiation (**f–h**), precipitation (**n–p**), and soil moisture (**r–t**). For comparison, the absolute values of the control experiment are shown (**a**,**e**,**i**,**m**,**q**). Regions without data correspond to those with no sunlight or the ocean. The scale for the no ejection case is different from the others. The BC emission in the no ejection case is mainly from biomass burning.

**Figure 4:**
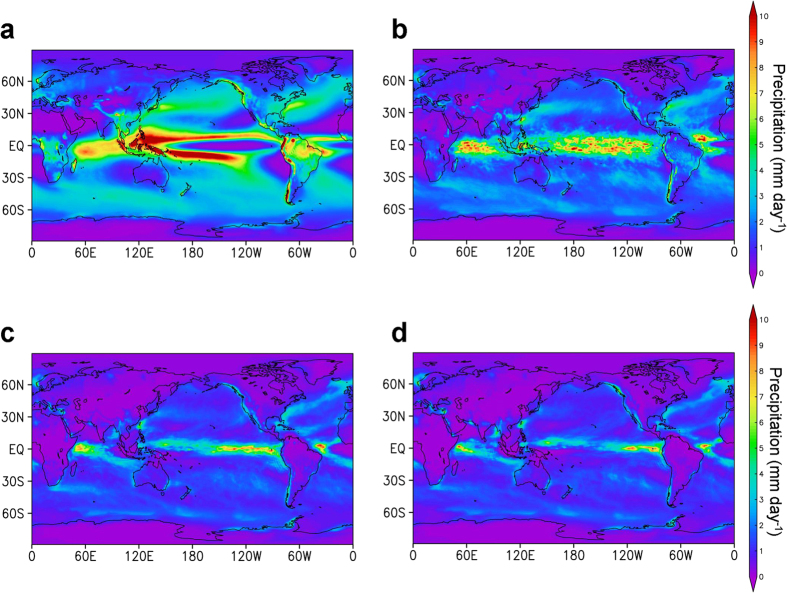
Global distributions of precipitation before and after the black carbon (BC) injection. (**a–d**) The 30-year average precipitation for the no ejection case (the pre-industrial climate conditions in the control experiment) (**a**), mean precipitation for 2 years from 6 months after the impact for the 500-Tg (**b**), 1500-Tg (**c**), and 2600-Tg (**d**) BC scenarios calculated by the climate model. The precipitation substantially decreased due to the impact in the 15°N to 15°S region on land, resulting in desert-like climates. The figure was created using the Grid Analysis and Display System (GrADS) Version 2.0 (http://www.iges.org/grads/).

We performed global climate model calculations to quantify the climate change caused by BC (equivalent to soot) injection for three quantities of BC (see Methods). The BC ejected into the stratosphere spread around the globe within a few months and was gradually deposited on the surface over the following five years (Fig. 2). BC in the stratosphere efficiently absorbs solar radiation and reduces the sunlight reaching the Earth’s surface, which leads to heating of the stratospheric atmosphere and cooling of the tropospheric atmosphere (Supplemental Fig. 2). The sudden cooling of the surface temperature weakened the hydrologic cycle, resulting in an abrupt and significant decrease in precipitation (Fig. 2). In the 500-Tg BC ejection scenario, the global-averaged model results indicated an abrupt decrease (by 50–60%) in sunlight just after the impact, with shortwave radiation gradually recovering over the following 10 years (Fig. 2). The blocking of sunlight resulted in a decrease in temperature at Earth’s surface, a 6–9 °C cooling that lasted for two years on land, and a 45–70% decrease in precipitation on land over the same period (Fig. 2). In the 1500- and 2600-Tg BC ejection cases, abrupt decreases (by 80–85% and 85–90%) in sunlight led to 10–16 °C and 10–18 °C cooling that lasted for three and five years on land and 55–80% and 60–85% decreases in precipitation on land over the same periods, respectively (Fig. 2). The temperature and precipitation gradually recovered within 10 years after the impact and the recoveries decreased over the following years. We found that these climate changes strongly depended on latitude in every BC ejection scenario (Fig. 3). The temperature cooling was dominant in the mid-high latitude regions due to the presence of large amounts of BC in the stratosphere in those regions. On the other hand, the cooling was milder in the low latitude regions. However, precipitation substantially decreased on land in those regions over several years after the impact (Figs 2, 3, 4). Table 1 lists the averaged lowest monthly paleotemperature and precipitation over land at 15° latitude intervals for January and July after the impact in the 500-, 1500-, and 2600-Tg BC ejection scenarios. We estimated the paleotemperature from the Maastrichtian land surface air temperature using BESTGUESS and BARESOIL by Upchurch *et al*.^40^ and the cooling temperatures from the impact estimated in the climate model calculations. We also estimated the precipitation from the late Cretaceous land precipitation using the GENESIS (v2.0) data^41^ and the decreasing land precipitation from the impact estimated in the climate model calculations. Based on the simulations of modern and Cretaceous climates by Bush and Philander^42^, we assumed that the geographical setting and presence of an ice sheet in the modern climate would not have significant effects relevant to the cooling temperatures between 60°N and 60°S.

### Seawater temperature change

**Figure 5:**
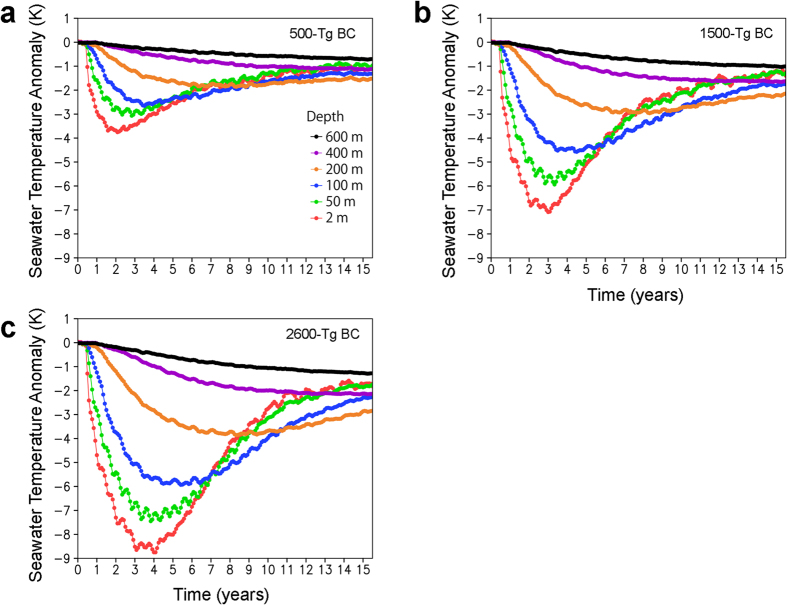
Seawater temperature changes caused by the black carbon (BC) injection. (**a–c**) Changes in the global averages of seawater temperature at 2-m, 50-m, 100-m, 200-m, 400-m, and 600-m water depths for the 500-Tg (**a**), 1500-Tg (**b**), and 2600-Tg (**c**) BC scenarios calculated by the climate model. Monthly anomalies from the control experiment (no ejection scenario) are shown. The 30-year global averages of seawater temperature at 2-m, 50-m, 100-m, 200-m, 400-m, and 600-m water depths in the control experiment were 293, 292, 290, 287, 283, and 280 K, respectively. The regions with seawater temperatures below zero at the 2-m water depth in the control experiment were excluded for the estimation of the anomalies and the 30-year averages to exclude the sea ice area.

**Figure 6:**
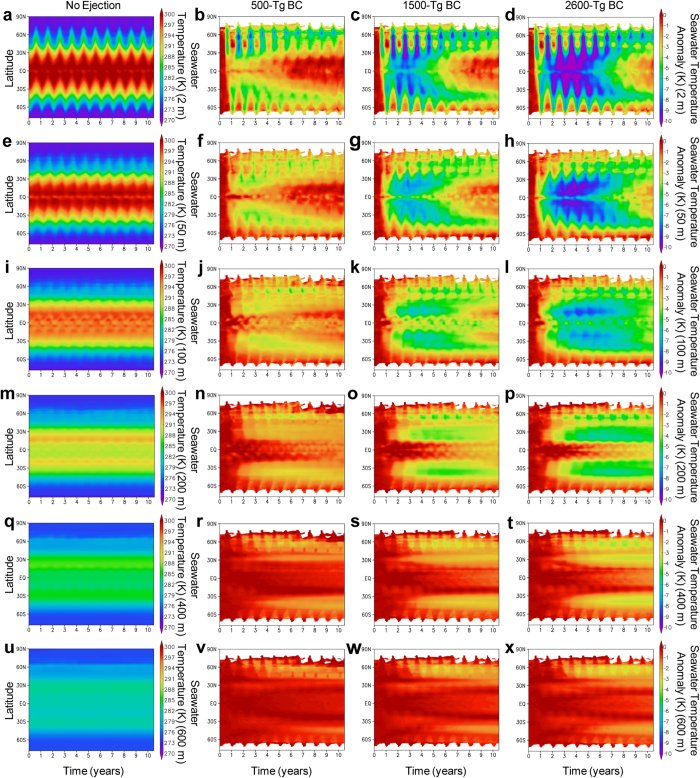
Meridional distributions of pre-industrial seawater temperature and seawater temperature changes caused by the black carbon (BC) injection. (**a–x**), Time–latitude cross-sections of zonal mean changes in seawater temperature at 2-m (**a–d**), 50-m (**e–h**), 100-m (**i–l**), 200-m (**m–p**), 400-m (**q–t**), and 600-m (**u–x**) water depths for the no ejection (control) (**a**,**e**,**i**,**m**,**q**,**u**), 500-Tg (**b**,**f**,**j**,**n**,**r**,**v**), 1500-Tg (**c**,**g**,**k**,**o**,**s**,**w**), and 2600-Tg (**d**,**h**,**l**,**p**,**t**,**x**) BC scenarios calculated by the climate model. Monthly anomalies from the control experiment are shown for the three BC scenarios (**b–d**,**f–h**,**j–l**,**n–p**,**r–t**,**v–x**). The regions with seawater temperatures below zero at the 2-m water depth in the no ejection (control) scenario were excluded for the estimation of the anomalies to exclude the sea ice area. For comparison, the absolute values of the control experiment are shown (**a**,**e**,**i**,**m**,**q**,**u**). Regions without data correspond to the sea ice area. The scale for the no ejection scenario is different from the others.

The reductions in surface shortwave radiation due to the BC ejection led to cooling of the ocean on a global scale (Fig. 5). Although the response time of the seawater cooling after the impact was slower than that of the surface air temperature and precipitation over land (Fig. 2), the seawater temperature largely decreased in the surface waters (0–100 m depths, e.g., up to 4 °C, 7 °C, and 9 °C cooling for the 500-, 1500-, and 2600-Tg BC ejection scenarios, respectively, at 2-m water depth in 2–4 years). The temperature reductions at deeper water depths (>200 m) were much slower and smaller than those at surface water depths (e.g., within 1 °C cooling at a 600-m water depth for the three cases in >15 years). The maximum cooling of surface waters appeared 2–6 years after the impact, and the cooling recovered gradually, taking more than a decade. Table 2 lists the monthly averaged lowest seawater temperatures at each water depth at 15° latitude intervals for January and July after the impact in the 500-, 1500-, and 2600-Tg BC ejection scenarios using the Maastrichtian ocean surface air temperature^40^ and the model calculations (Fig. 6).

## Discussion

### Origin of soot

We assumed that the combusted PAHs in the coarse-ejected sediments were transported from the troposphere, and those in the superjacent fine sediments were sourced from the stratosphere and land. The high coronene/TOC, high Cor/(Cor + BeP + Bpery), and low CPI values of the *n*-alkanes in the coarse-ejecta beds and the Ir layer in Haiti and the similar Cor/(Cor + BeP + Bpery) values in Haiti and Spain (Fig. 1 and Supplemental Tables 2 and 3) indicate the high temperature combustion of oil and the same source for the PAHs from the troposphere and stratosphere, suggesting that the impact of the asteroid into the oil field launched an enormous cloud of smoke into the stratosphere from the Chicxulub crater.

Dead organisms and crude oil present at the end of the Cretaceous were mixed in the ejecta. Cretaceous carbonate rocks and K/Pg impact breccia from a borehole in the Chicxulub crater contain oil shales^43^, but most of the crude oil in the target rocks at Chicxulub was lost during the impact, leaving no direct evidence and no information on the original amounts of crude oil and other organic matter. The K/Pg impact breccias in the Cantarell Field, which is the largest oil field in Mexico and located in the vicinity of the crater, contain massive amounts of crude oil derived from the Late Jurassic (i.e., 90 million years older than the asteroid impact)^44^. This is indirect evidence of the presence of very large amounts of crude oil in the target rocks at the end of the Cretaceous. The crude oil was combusted during the impact, as evidenced by carbon cenospheres from Canada, Denmark, and New Zealand^28^.

### Consistency between the geological record and the model results

**Figure 7:**
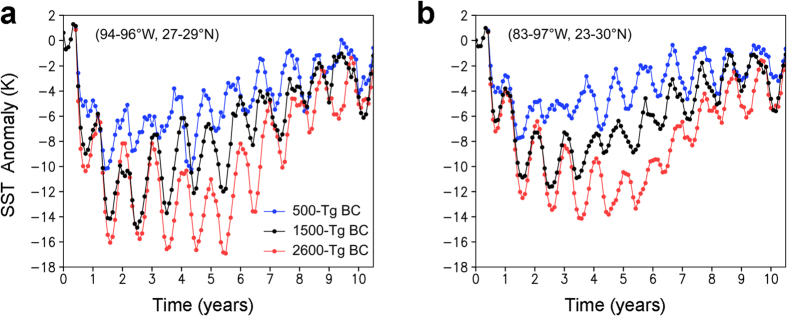
Changes in sea surface temperature (SST) caused by the black carbon (BC) injection. (**a**,**b**) Changes in the average SST values at the proximal site in the Gulf Coast (94–96°W, 27–29°N) (**a**) and over the Gulf of Mexico (83–97°W, 23–30°N) (**b**) for the 500-Tg (blue), 1500-Tg (black), and 2600-Tg (red) BC scenarios calculated by the climate model. Monthly anomalies from the control experiment (no ejection scenario) are shown. The 30-year global averages of the corresponding SST in the control experiment were 295 K and 297 K, respectively.

There was consistency between the geological record and the model results on two events: 1) decreasing temperature for the same area assuming ejections of 500–2600 Tg BC by the impact and 2) drought-devastation of land plants at low latitudes. The consistency supports the use of the model results. The geological sea surface temperature (SST) record in the marlstone just above the coarse ejecta at a proximal site on the Gulf Coast showed a rapid 7 °C cooling followed by a gradual increase in SST coinciding with the second input of Ir from the stratosphere (the first Ir concentration at the top of the coarse sedimentary rocks is thought to have been derived from the troposphere)^44^. The 7 °C SST cooling is within the ranges of the model results, that is, 7 °C and 5 °C average SST cooling at the site and over the Gulf of Mexico, respectively, during the three years after the impact, followed by a gradual increase in the 500-Tg BC ejection scenario (Fig. 7). In the 1500- and 2600-Tg BC ejection scenarios, the corresponding SST coolings were 10 °C, 8 °C, 11 °C and 9 °C, respectively (Fig. 7). Land plant devastation in the 15°N to 15°S region estimated using the model calculation (see “Drought and extinction or survival on land”) was observed in the increase of δ^13^C of C29 and C31 long-chain *n*-alkanes during the minima of δ^13^C of C16 short-chain *n*-alkanes in Haiti located at 15°N (Fig. 1).

### Cooling and extinction or survival on land

For the estimated land surface air temperatures, dinosaurs and tropical plants^45^ could have survived only at the low latitudes in the 500-Tg ejection case, may have survived only in the equatorial area in the 1500-Tg ejection case, and could not survive at any location in the 2600-Tg ejection case on land (Table 1; survival only in summer means extinction). For dinosaurs, this assumes a seasonal migration and a 15 °C monthly average temperature as the lower limit for survival based on correlations of the diversity of dinosaurs vs. paleolatitude^46^ and the summer land surface air temperature vs. paleolatitude^40^. For example, the postulated 15 °C monthly average temperature with a seasonal migration can account for dinosaur bones found in the North Slope of Alaska^47^, because the average paleotemperature was 12–15 °C in July (Table 1). The temperature limit is consistent with the distribution of recent crocodilians, which are taxonomically and ecologically related to dinosaurs^48^, and survive only in areas where the winter temperature in January or July is >15 °C without seasonal migration^49^. Although small mammals and reptiles could have lived underground where it is warmer, the dinosaurs did not. The different habitats of the dinosaurs and small mammals and reptiles would also have been key factors in determining their extinction or survival^50^.

### Cooling and extinction or survival in oceans

Late Cretaceous (Campanian-Maastrichtian) ammonites inhabited 16–32 °C annual mean temperature seawater based on oxygen isotopes (δ^18^O) of ammonite shells^51^,^52^,^53^,^54^. These ammonites should have inhabited between 200–100-m and 0-m water depths. Inoceramid bivalves were shallow dwellers^52^ and became extinct at the K/Pg boundary. Both shallow- and deep-dwelling planktonic foraminifera inhabited the surface water (<~100-m water depths) during at least a portion of their lifetimes (<1 year). These extinct marine organisms in the surface water likely suffered from low seawater temperatures resulting from cooling of 2–7 °C, 3–10 °C, and 3–11 °C for the 500-, 1500-, and 2600-Tg BC ejection cases, respectively, within 2–5 years after the impact (Supplemental Table 4). The habitat depths of ammonites changed with ontogenetic stage (juveniles, middle phase, and adults)^55^. The highest temperature during ontogeny may be used to classify ammonites into three groups: cool water type (16–20 °C), mild water type (21–25 °C), and warm water type (26–32 °C). These temperatures are supported by oxygen isotope data of late Cretaceous ammonite shells^51^,^52^,^53^,^54^. The warm water ammonites could not survive after the impact (Table 2). The mild water ammonites could not survive after the impact in the 1500-Tg and 2600-Tg BC cases. The cool water ammonites survived upon migration to shallower parts in these cases. This is consistent with evidence that those ammonites that survived briefly into the Paleocene, and the nautilids that survive to the present day, were significantly more widely distributed than were those that disappeared at the end of the Cretaceous^8^. The cooling of seawater could have disturbed the physiology and food web of the shallow marine dwellers, such as planktonic foraminifera, ammonites, inoceramids, and marine reptiles, which could have led to their extinction. Deep dwellers such as nautilids, sepia, and benthic foraminifera survived the K/Pg crisis^8^,^55^,^56^,^57^,^58^ likely because of the smaller changes in seawater temperature in intermediate and deep waters (Figs 5 and 6). Cooling due to stratospheric soot aerosols could have caused the extinction of shallow dwellers that inhabited 100–0-m water depths (Table 2). Temperature minima occurred within 3–6 years after the impact in 100-m water depths in oceans but within 1–3 years on land. Extinctions in oceans should have followed land plant devastation and extinction of the dinosaurs. In fact, fossil records show that the widely distributed ammonites briefly survived after the fall of Ir^7^.

### Sunlight and extinction/survival

The global weak sunlight within 1 month to 2 years after the impact (Figs 2 and 3) was sufficient for photosynthesis to occur on land and the sea surface^59^ for the three BC scenarios but insufficient in oceans at >~50-m water depths (the 50-m water depth received 0.2–1% of the sunlight on sea surface before the asteroid impact, which corresponds to the limit for photosynthesis)^60^, which caused a cessation of photosynthesis and a decrease in marine productivity evidenced by calcareous nannofloral assemblages^61^ and resulted in a decrease in marine animal species. The deep-dwelling cool-water ammonites first suffered from the cessation of photosynthesis and then from the decrease in seawater temperature. They may have survived the temperature change but not the lack of photosynthesis and resulting lack of a food source. Furthermore, soot from wild fires (~70,000 Tg BC)^21^,^27^ and from impact ejecta (~1500 Tg BC) fell into the oceans near the impact site and in high precipitation areas and was then carried by ocean currents, which may have induced an additional reduction in sunlight leading to the cessation of photosynthesis in shallower waters at <50-m water depths. Sulfuric acid rain may have also contributed to the marine extinction^20^. Sulfuric acid rain near the impact site was carried by ocean currents, which may have resulted in a global decrease in pH in the surface waters within several years after the impact.

### Drought and extinction or survival on land

The abrupt, significant decrease in precipitation obtained by the model calculations could apply to the paleoclimate because the distribution and amounts of precipitation in the late Cretaceous do not differ substantially from those of the present day^41^,^42^ (Fig. 3). In the 15°N to 15°S region for the 500-Tg, 1500-Tg, and 2600-Tg ejection cases, annual mean precipitation over the land decreased by 75% (to 1.1 mm/day), 93% (0.3 mm/day), and 94% (0.3 mm/day), which lasted for 1.5, 2.5, and 3.5 years, respectively (Fig. 3, Table 1). The low precipitation, similar to that of the Sahara Desert, occurred in all areas over the land in the 15°N to 15°S region for the 1500-Tg and 2600-Tg BC ejection scenarios (Fig. 4), suggesting desert-like precipitation conditions over those areas after the impact. Soil moisture near the surface decreased by up to 40% for the 500-Tg ejection scenario and 50% for the 1500-Tg and 2600-Tg ejection scenarios in the equatorial zone (Fig. 3), which may have caused significant damage to the tropical herbaceous and broad-leaved vegetation, except for tropical broad-leaved evergreens^45^, leading to further loss of soil moisture and vegetation. The decreasing cover and size of vegetation patches resulted in further losses of soil and water, which led to further vegetation loss^62^. The herbivorous dinosaurs ate the decreased number of plants, resulting in the disappearance of vegetal food, similar to overgrazing leading to desertification today^63^,^64^, which could have led to the extinction of the dinosaurs. Survival of freshwater-dwelling vertebrates such as crocodilians is dependent the availability of food, such as birds, mammals, fish, reptiles (turtles, snakes), amphibians, and macroinvertebrates and the base of the food webs being plant detritus^6^,^65^. The riverine communities could have survived because their organic carbon was derived from detritus^6^,^65^ and water sourced from the middle latitudes had sufficient precipitation.

### Summary

A soot ejection of 500 Tg could not have caused extinction of the dinosaurs and ammonites, while an ejection of 2600 Tg could have induced extinction of the dinosaurs, crocodilians, and warm-mild water ammonites. Dinosaurs and warm-mild water ammonites could have become extinct, while the crocodilians survived in the 1500-Tg soot ejection case (Tables 1 and 2). The diminishing photosynthesis and food-web collapse in oceans may have killed the cool-water deep-dwelling ammonites. Therefore, a soot ejection of ~1500 Tg seems to be sufficient for the mass extinction and survival that occurred at the K-Pg boundary, as supported by the correspondence between the model results and the geological record. Our results show that the stratospheric aerosols did not induce darkness, in contrast to previous assumptions. The surface cooling was milder than previously thought. Sufficiently colder climates at mid–high latitudes caused extinction of the dinosaurs and crocodilians at mid–high latitudes; the drought accompanied by mild cooling at low latitudes on land led to extinction of the dinosaurs and allowed survival of the crocodilians at low latitudes. The mild cooling with diminishing photosynthesis in the oceans could have caused extinctions of marine organisms such as ammonites, inoceramids, and most planktonic foraminifera, while allowing the survival of deep-sea organisms. The sufficiently colder climates at mid–high latitudes and drought with milder cooling at low latitudes on land, as well as the limited cessation of photosynthesis in global oceans occurred a few months to 2 years after the impact, followed by surface water cooling in global oceans within 2 to 6 years after the impact. The climate change could have led to the terrestrial extinctions within a few years, followed by the marine extinctions over several years. If darkness (no sunlight) had occurred for a few years after the impact, the resulting low temperatures would have caused extinction of the crocodilians, birds, and mammals. The cooling would have been mild to allow their survival on land. The stratospheric soot-aerosol can explain the extinction and survival pattern at the K/Pg boundary. Our results show that rapid global climate change can play a major role in driving extinction.

## Methods

### Sedimentary organic molecule analyses

Approximately 50–100-g hand-sized rock specimens were powdered for each sample after the removal of any apparent surface contamination. The powdered samples were extracted for 48 h using a Soxhlet apparatus and a dichloromethane:methanol mixture (7:1 v/v). The extracts were dried over Na_2_SO_4_ and concentrated by evaporation under reduced pressure. The concentrated extracts were separated into nine fractions on a silica gel column (0.6 g silica, 63–200 μm) by elution using the following solvents: 2 mL *n*-hexane (F1a), 4 mL *n*-hexane (F1b), and 3 mL *n*-hexane/toluene 3:1 v/v (F2). The aromatic hydrocarbon fraction (combination of F1b and F2) from each extract was analyzed by gas chromatography (GS)–mass spectrometry. Identification of the organic compounds was performed using a gas chromatograph (model 6893; Agilent, Santa Clara, CA, USA) interfaced to a mass-selective detector (model 5973; Agilent).

The stable carbon isotope ratios of each compound in the F1b and F2 fractions were also determined using a GC–isotope ratio mass spectrometer system (Trace GC Ultra, Delta-V advantage; Thermo Fisher Scientific, Waltham, MA, USA). Detailed descriptions of the stable carbon isotope ratio are given in the Supplementary Information.

### Model calculation

We used a coupled atmosphere–ocean global climate model developed at the Meteorological Research Institute, MRI- CGCM3^66^, which consists of the atmospheric general circulation model coupled with the land surface model, the ocean general circulation model using the sea ice calculation, and the aerosol chemical transport model. The model can estimate the climate changes caused by aerosols based on the pre-industrial modern climate conditions and current geographical settings. We performed three 15-year experiments using BC ejection due to the asteroid impact (500-Tg, 1500-Tg, and 2600-Tg BC cases) and a 30-year control experiment with no ejection. We evaluated the climate response due to the BC ejection by subtracting the monthly climatology (30-year mean) of the control experiment from the monthly mean results of the other experiments. Detailed descriptions and evaluations of the model calculations are given in the Supplementary Information.

## Additional Information

**How to cite this article**: Kaiho, K. *et al*. Global climate change driven by soot at the K-Pg boundary as the cause of the mass extinction. *Sci. Rep.* **6**, 28427; doi: 10.1038/srep28427 (2016).

## Supplementary Material

Supplementary Information

## Figures and Tables

**Table 1:**
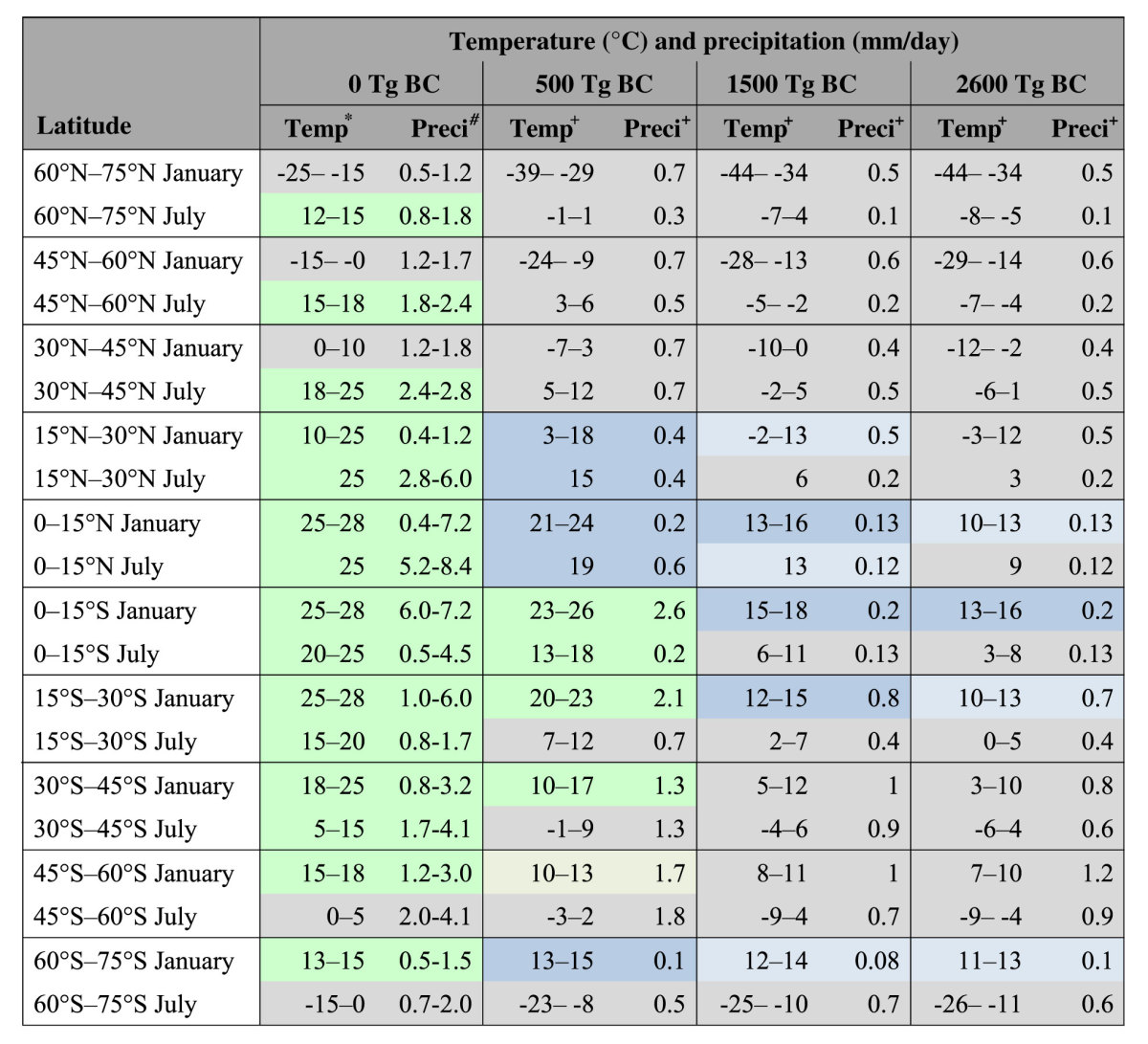
Monthly averaged surface air temperatures and precipitation over land in January and July at 15° latitude intervals before and after the soot ejection from the impact accompanied by the estimated latitudinal areas and seasons of survival and extinction of the dinosaurs and crocodilians.

^*^Latest Cretaceous monthly mean land surface air temperature after average values from the BESTGUESS and BARESOIL simulations^40^.^#^Late Cretaceous monthly mean land precipitation from the GENESIS (v2.0) data^41^.^+^Estimates by subtracting the maximum decreasing land surface air temperatures and land precipitation of the climate model calculations of this study (Fig. 3) from the latest Cretaceous land surface air temperature and the late Cretaceous land precipitation (average within the range), respectively. The areas of survival of most of the dinosaurs are shown in green (>15 °C, >1.0 mm/day). The areas of survival of the crocodilians are shown in green and blue (>15 °C). Pale green and blue areas indicate the areas of survival considering a 2 °C error (>13 °C). Low precipitation (<0.1 mm/day) during half year corresponds to areas of survival. Gray areas indicate those where the dinosaurs and crocodilians did not survive.

**Table 2:**
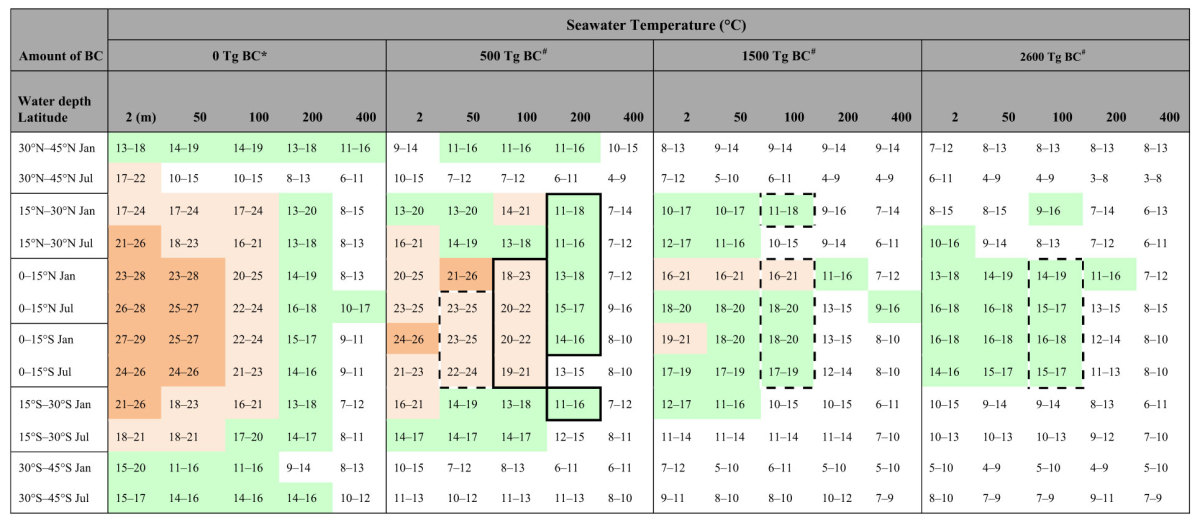
Monthly averaged lowest seawater temperatures at 2- to 400-m water depths in January and July at 15° latitude intervals before and after soot ejection from the impact and habitable latitudinal and water-depth areas and seasons of the extinct marine cephalopoda, ammonites.

*Estimates by subtracting the difference between surface air temperature and seawater temperatures at each water depth of the monthly climatological temperature of the control experiment (30-year mean in the pre-industrial condition) from the latest Cretaceous monthly mean surface air temperature over the ocean after average values from the BESTGUESS and BARESOIL simulations^40^.^#^Estimates by subtracting the maximum decreasing seawater temperatures at each water depth in each BC ejection scenario of the climate model calculations of this study (Supplemental Table 4) from the latest Cretaceous monthly mean seawater temperature at each water depth (see 0 Tg BC column). Seawater temperature data where late Cretaceous ammonites (Campanian-Maastrichtian) inhabited can be divided into three groups: 16–20 °C (cool water type: green), 21–25 °C (mild water type: pale orange), and 26–32 °C (warm water type: orange) (see text). Solid-framed areas show the survival area estimated. Broken-framed areas show that migration was required for survival. The seawater temperatures at 200-m and 400-m water depths should be slightly higher, because the latest Cretaceous deep water (>1000-m water depths) temperature was ~10 °C higher than that of the present day.
