# Supplementary material for: Global climate change driven by soot at the K-Pg boundary as the cause of the mass extinction
Source: Sci Rep. 2016 Jul 14;6:28427. doi: 10.1038/srep28427 (PMC4944614; doi:10.1038/srep28427)
Supplement: Supplementary Information [file srep28427-s1.docx]

**Supplementary Information**

**Global climate change driven by soot at the K-Pg boundary as the cause of the mass extinction**

**Kunio Kaiho^1^, Naga Oshima^2^, Kouji Adachi^2^, Yukimasa Adachi^2^, Takuya Mizukami^1^, Megumu Fujibayashi^3^, Ryosuke Saito^1^**

^1^Department of Earth Science, Tohoku University, Sendai, Japan. ^2^Meteorological Research Institute, Tsukuba, Japan. ^3^Ecological Engineering Laboratory, Tohoku University, Sendai, Japan. Correspondence and requests for materials should be addressed to K.K. ([Kaiho@m.tohoku.ac.jp](mailto:Kaiho@m.tohoku.ac.jp))

**Materials and Methods**

**Geological Setting.** Organic molecular data represent analyses of sedimentary rock samples from the following proximal K/Pg and distal sections.

We examined a proximal K/Pg section, “the stratotype section” at Beloc in Haiti located approximately 700 km south of Chixulub at the end-Cretaceous (Fig. 1)^68^; coarse-ejecta beds of 60-cm thick calcareous sandstones that contain microspherules and impact glass directly overlie the uppermost Maastrichtian marlstones, which contain many large Maastrichtian planktonic foraminifera^24,25^ (Supplemental Fig. 1). The coarse-ejecta beds containing microspherules formed by the impact are overlain by a silty very fine sandstone of 10-cm thickness and a marlstone layer of 12-cm thickness (Fig. 1). Within the marlstone, an iridium (Ir) accumulation^24^ sourced from the asteroid is contained in a 1–2 cm rust-orange calcareous clay layer, referred to as the “red layer” (or “fine ejecta”). The marlstone is overlain by limestones that contain small Danian planktonic foraminifera^24,25^ (Supplemental Fig. 1). The low concentration of terrestrial plant organic molecules (Fig. 1) and the common occurrence of planktonic foraminifera in the coarse ejecta (Supplemental Fig. 1) indicate a marine source rather than a terrestrial origin for the coarse ejecta.

The Caravaca K/Pg section (Fig. 1) is located in the Betic Cordillera of southeastern Spain (38°04′35″N, 1°52′40″W). Marlstones of Cretaceous age are lithologically separated from marlstones of Paleogene age by a 7–10 cm thick, dark, clay–marl bed (the boundary clay layer). Within the boundary clay layer, a 1–2 mm, rust-orange, basal layer referred to as the “red layer” (or “fallout lamina”) contains the iridium (Ir) anomaly^69^ and is underlain by a 3 mm greenish transition layer. Here, the base of the red layer is defined as the K/Pg boundary. The Caravaca section represents paleowater depths of 200–1000 m^70,71^.

**Sedimentary Organic Molecules.** A gas chromatograph (model 6893: Agilent, Santa Clara, California, USA) interfaced to a mass-selective detector (MSD: model 5973, Agilent) was operated with an ionizing-electron energy of 70 eV, and scanned from *m/z* 50 to 550, with a scan time of 0.34 s. A fused silica HP-5MS capillary column (30 m, 0.25 mm i.d., 0.25 μm film thickness) was used, with helium as the carrier gas. Samples were injected at 50°C and held at that temperature for 1 min. Then the temperature was raised to 120°C at a rate of 30°C/min, then to 310°C at a rate of 5°C/min, and finally held constant for 20 min.

CPI ={[(C25 + C27 + C29 + C31 + C33)/ (C24 + C26 + C28 + C30 + C32)] +

[(C25 + C27 + C29 + C31 + C33)/ (C26 + C28 + C30 + C32 + C34)]} /2

**Stable Carbon Isotope Ratio.** Helium was used as the carrier gas at a constant flow rate of 0.8 mL/min. The temperature of the inlet and combustion interface was maintained at 260°C and 1030°C, respectively. The column and oven condition was the same as that for the GC-MS analysis (see above). Peak identification was conducted according to retention time by comparison with commercial standard mixtures. Carbon stable isotope ratios were expressed relative to Vienna Pee Dee Belemnite (VPDB). The instrument precision of the measurement system was ±0.1‰. We measured each sample twice, when possible, to produce a more reliable value based on the shape of the peak and the amount shown by mV in Figure 1. The carbon isotopic compositions of each organic molecule are shown in Table 1. The data in Figure 1 were selected from those data and were from samples having sharper peak shapes. When there was no difference in the peak shape, we used data from samples having a higher amount shown by mV.

**Amount of CO_2_.**  The asteroid impact emitted a large amount of CO_2_ through evaporating carbonate. We calculated the amount of CO_2_ to be 729 Gt (an increase of 91 parts per million by volume (ppmv) as a global average) from a calculation by Pierazzo et al.^72^ by assuming a 10 km diameter for the asteroid and a density of 3.32 g/cm^3^.

**Amount of Soot in the stratosphere.** We assumed that soot was equivalent to black carbon (BC), and therefore used its optical and physical properties in the climate model.

The amounts of soluble Cor + BeP + Bpery detected in this study in the K/Pg fine ejected deposits at Beloc (ejected into the stratosphere) and the K/Pg ejected layer at Caravaca were 8.5 and 3.3 ng/cm^2^, respectively (Fig. 1 and Supplemental Tables 2 and 3). We used 6 ng/cm^2^ (the mean value of 8.5 and 3.3 ng/cm^2^) as the average value of BC ejected into the stratosphere.

Soluble coronene was not likely to have been produced during diagenesis^73^. PAHs with four or more aromatic rings were below the detection limit in the bitumen, but were present in kerogens in shales from 2.5 billion years ago^74^. Most of the other PAHs (up to 99%) were in kerogens and the remainder were in bitumen^75^. These suggest that Cor + BeP + Bpery were installed in high-molecular-weight substances during the formation of the kerogen, and were accompanied by a slight production of soluble five- to six-ring PAHs during the diagenesis, resulting in a decrease in the amount of soluble Cor + BeP + Bpery. However, the rate of the decrease for the K-Pg samples studied is probably less than 90%, although the exact value is unknown. The amounts of dissolved Cor + BeP + Bpery were assumed to be 12, 36, and 62 ng/cm^2^ at 66 Ma, which correspond to a decrease of 50, 83, and 90% in soluble organic molecules, respectively, during the 66 million years.

The soot is comprised of the PAHs^76^. The amount of soot that was distributed globally throughout the stratosphere was 0.04, 0.12, and 0.21 mg/cm^2^ (200, 600, and 1000 Tg on the Earth’s surface) in the K/Pg Ir layer using 0.3 mg/g for the content of Cor + BeP + Bpery in diesel soot^77^. The ratio of BC/(Cor + BeP + Bpery) is 1.6 × 10^4^ (R = 0.98, n = 15) in the Permian mass extinction horizon (Bed 25 to 30 of the Meishan section, China)^78^, which results in an amount of 0.10 mg/cm^2^ soot in the stratosphere at the K/Pg boundary; this value is consistent with our estimates.

A model calculation of the distribution of the ejecta by an impact of an asteroid 10 km in diameter showed that 62% of the ejecta reached an altitude of 0 to 10 km, 20% reached 10–30 km, and 18% reached 30–50 km^78^. Thus, we estimated 500, 1500, and 2600 Tg BC as the total soot ejecta.

**Model Calculation.** We used a coupled atmosphere–ocean global climate model, MRI-CGCM3^66^. The details of this model are described elsewhere^66^. The aerosol model is interactively coupled with the atmospheric model, which enables an explicit representation of the effects of aerosols on the climate system. The MRI-CGCM3 model has previously been evaluated against observations, including reanalysis, and can reproduce the overall present-day mean climate^66^. The model contributed to the Fifth Assessment Report of the Intergovernmental Panel on Climate Change^79^ by participating in the Climate Model Intercomparison Project phase 5 [CMIP5^80^].

The MRI-CGCM3 model was run at horizontal resolutions of approximately 120 km (TL159) and 180 km (TL95) for the atmospheric and aerosol models, respectively, with 48 vertical layers for both models from the surface to a model top of 0.01 hPa (approximately 80 km in altitude), which covers all of the stratosphere. The land surface model employed 14 soil layers within a total depth of 10 m, with finer intervals near the surface (6 layers above a depth of 50 cm). The oceanic portion of the model has a 1° × 0.5° longitude–latitude resolution and employs 50 vertical levels with an additional bottom boundary later. The ocean surface layer is 4 m thick and the upper layers above a water depth of 1000 m are resolved by 30 layers. The initial state used in the model calculations was taken from the CMIP5 pre-industrial control experiment conducted as an MRI-CGCM3 calculation for several hundred years and exhibited a sufficient stable equilibrium state, without climate drifts. All external forcing agents including the emissions from anthropogenic and biomass burning sources were fixed for conditions in the year 1850 from the Representative Concentration Pathways database^81^, following the CMIP5 pre-industrial control experiment. The optical properties in the solar and terrestrial spectral range of the atmospheric aerosols were calculated on the basis of microphysical data such as the size distribution and spectra refractive index by the software package Optical Properties of Aerosols and Clouds (OPAC)^82^.

We performed three 15-year experiments with the BC ejection due to the asteroid impact (500-Tg, 1500-Tg, and 2600-Tg BC cases) and a 30-year control experiment with no ejection from the same equilibrium initial state using pre-industrial climate conditions on January 1. The BC was ejected into one column of the model grid box at (21°N, 90°W), at the Yucatan Peninsula in the current geographical setting, over a one-day period on June 1 in the first year. The 62, 20, and 18% BC ejections were vertically distributed over ranges of 0 to 10, 10 to 30, and 30 to 50 km altitude within the column, respectively, and were evenly spread within each altitude range. We focused on the three-dimensional spread of the BC ejection by atmospheric transport for one month after the asteroid impact and began calculations of the aerosol effects on atmospheric radiation from July 1. We ignored the initial destructive effects of the asteroid itself and the short-term local extreme atmospheric changes for the first month of the impact because of the limitations of the current model. In addition to the BC ejection, we considered the impact of the CO_2_ injection over the globe (91 ppmv increase in the global average) for the three BC cases.

We also performed two additional 10-year sensitivity experiments for the 1500-Tg BC ejection case. One was a no-CO_2_ ejection experiment, which excluded the CO_2_ ejection from the 1500-Tg BC case, to quantify the enhancement of the greenhouse effect caused by the CO_2_ injection following the impact. Comparison with the 1500-Tg BC case showed that the change in surface air temperature over land caused by the CO_2_ injection was negligible for the first six years after the impact, with slight increases (~0.5 K in global average) over the following four years (Supplemental Fig. 3), indicating that CO_2_ levels had a negligible effect on climate change within a 10-year time scale. The second experiment considered a larger BC particle size distribution, using that observed by aircraft measurements above the remote Pacific, from the near-surface to the lower stratosphere (i.e., mode radius of 43.7 nm and geometric standard deviation of 1.64 for the lognormal number size distribution)^83^, with all other settings the same as for the 1500-Tg BC case. The BC size distribution of the OPAC dataset (i.e., 11.8 nm and 2.00, respectively)^82^ used in MRI-CGCM3, was generally smaller than the observed size distributions in the present-day atmosphere^22^. Compared to the 1500-Tg BC case, the larger BC size case gave a smaller BC loading in the atmosphere, resulting in a smaller amplitude of climate change (Supplemental Fig. 3). The comparison also showed that the climate change experienced in the larger BC size case recovered from the influence of the impact approximately one year earlier than for the 1500-Tg BC case. Although the exact particle size distribution emitted by the impact is unclear, the size sensitivity experiment suggested that possible uncertainties, due to the size distribution of BC particles, with regard to changes in the land surface air temperature and precipitation could be within 3 K and 0.3 mm day^-1^, respectively, as monthly global averages.

We used a climate model that was originally developed to evaluate the modern climate, to simulate the injection of BC and CO_2_ into pre-industrial climate conditions and current geographical settings. The end-Cretaceous climate is different from the pre-industrial climate, i.e., it was warmer with no ice sheet (higher CO_2_ content), resulting in the use of different initial values in this study. The injection of water vapor (~1400 Gt) due to the impact was not considered in the model calculation, but it is likely to have had little influence on the extinction event, because the effect on the climate would only become important after the aerosols were removed from the atmosphere, allowing solar radiation to again reach the surface^68^. The model does not include coagulation of aerosols. However, the lack of this process will not alter the conclusions, because the BC amounts were estimated from the globally distributed BC through the stratosphere. The model did not contain a carbon cycle feedback for plants; cooling led to a decrease in plant cover causing an increase in CO_2_ and then warming. This process has a long time lag, and thus could have had only a negligible influence on the rapid mass extinction (Supplemental Fig. 3).

Condensed five- to six-ring PAHs^84^, soot^43,85^, and Ir^1,2,4^ have been recorded in sedimentary rocks formed from the fine ejecta around the world. Sulfuric acid may have also been formed during the impact and deposited as acid rain at the K/Pg boundary^17,18^. This study considered only the soot (BC) ejected by the impact. The presence of other aerosol species (e.g., sulfuric acid or dust) in the stratosphere could have contributed to the enhancement of weak sunlight and cooling of the Earth’s surface over several years by scattering solar radiation. On the other hand, these aerosols would coagulate with BC particles in the stratosphere, increasing their particle size and resulting in a shorter atmospheric lifetime, which would weaken the cooling effect.

**References**

68. Olsson, R. K., Miller, K. G., Browning, J. V., Habib, D. & Sugarma, P. J. Ejecta layer at the Cretaceous-Tertiary boundary, Bass River, New Jersey (Ocean Drilling Program Leg 174AX). *Geology* **25,** 759–762 (1997).

69. Smit J. & Ten Kate W. G. H. Z. Trace element patterns at the Cretaceous–Tertiary boundary—consequences of a large impact. *Cretaceous Research* **3,** 307–332 (1982).

70. Coccioni, R. & Galeotti, S. K–T boundary extinction: geologically instantaneous or gradual event? Evidence for deep-sea benthic foraminifera. *Geology* **22,** 779–782 (1994).

71. MacLeod, N. & Keller, G. Comparative biogeographic analysis of planktonic foraminiferal survivorship across the Cretaceous/Tertiary (K/T) boundary. *Paleobiology* **20,** 143–177 (1994).

72. Pierazzo, E., Kring, D. A. & Melosh, H. J. Hydrocode simulations of the Chicxulub impact event and the production of climatically active gases. *J. Geophys. Res*. **103,** 28606–28625 (1998).

73. Hallaman, C. et al. Molecular signature of the Neoproterozoic Acraman impact event. *Organic Geochemistry* **41,** 111–115 (2010).

74. Brocks, J. J. et al. Release of bound aromatic hydrocarbons from late Archean and Mesoproterozoic kerogens via hydropyrolysis. *Geochim. Cosmochim. Acta* **67,** 1521–1530 (2003).

75. Frencha, K. L. et al. Reappraisal of hydrocarbon biomarkers in Archean rocks. *Proc. Natl. Acad. Sci. USA* **112,** 5915-5920. (2015).

76. Wang Z. et al. Quantitative Characterization of PAHs in Burn Residue and Soot Samples and Differentiation of Pyrogenic PAHs from Petrogenic PAHs-The 1994 Mobile Burn Study. *Environ. Sci. Technol.* **33,** 3100-3109 (1999).

77. Shen, W., Sun, Y., Lin, Y., Liu, D. & Chai, P. Evidence for wildfire in the Meishan section and implications for Permian–Triassic events. *Geochim. Cosmochim. Acta* **75,** 1992–2006 (2011).

78. Saito, T., Kaiho, K., Abe, A., Katayama, M. & Takayama, K. Hypervelocity impact of asteroid/comet on the oceanic crust of the earth. *International Journal of Impact Engineering* **35,** 1770–1777 (2008).

79. IPCC. *Climate Change 2013: The Physical Science Basis. Contribution of Working Group I to the Fifth Assessment Report of the Intergovernmental Panel on Climate Change* (eds Stocker, T. F. *et al*.) 1-1535 (Cambridge Univ. Press, 2013).

80. Taylor, K. E., Stouffer, R. J. & Meehl, G. A. An Overview of CMIP5 and the experiment design. *Bull. Am. Meteorol. Soc.* **93,** 485-498, doi:10.1175/BAMS-D-11-00094.1 (2012).

81. Vuuren, D. P. *et al*. The representative concentration pathways: an overview. *Clim. Change* **109,** 5–31 (2011).

82. Hess, M., Koepke, P. & Schult, I. Optical properties of aerosols and clouds: The software package OPAC. *Bull. Am. Meteorol. Soc.* **79,** 831–844 (1998).

83. Schwarz, J. P. *et al*. Global-scale black carbon profiles observed in the remote atmosphere and compared to models. *Geophys. Res. Lett.* **37,** L18812, doi:10.1029/2010GL044372 (2010).

84. Arinobu, T., Ishiwatari, R., Kaiho, K. & Lamolda, M. A. Spike of polycyclic aromatic hydrocarbons associated with an abrupt decrease in δ^13^C of a terrestrial biomarker at the Cretaceous-Tertiary boundary at Caravaca, Spain. *Geology* **27,** 723-726 (1999).

85. Wolbach, W. S. & Gilmour, I. Major wildfires at the Cretaceous-Tertiary boundary. In *Global catastrophes in Earth history* (eds Sharpton, B. & Ward, P.) *Geol. Soc. Am., Boulder, Spec. Pap.* **247,** 391-400 (1990).

**Supplemental Figure 1. Stratigraphic variation in grain size and planktonic foraminiferal species across the Cretaceous/Paleogene boundary in the Beloc section.** Planktonic foraminiferal data after Mizukami et al.^25^.


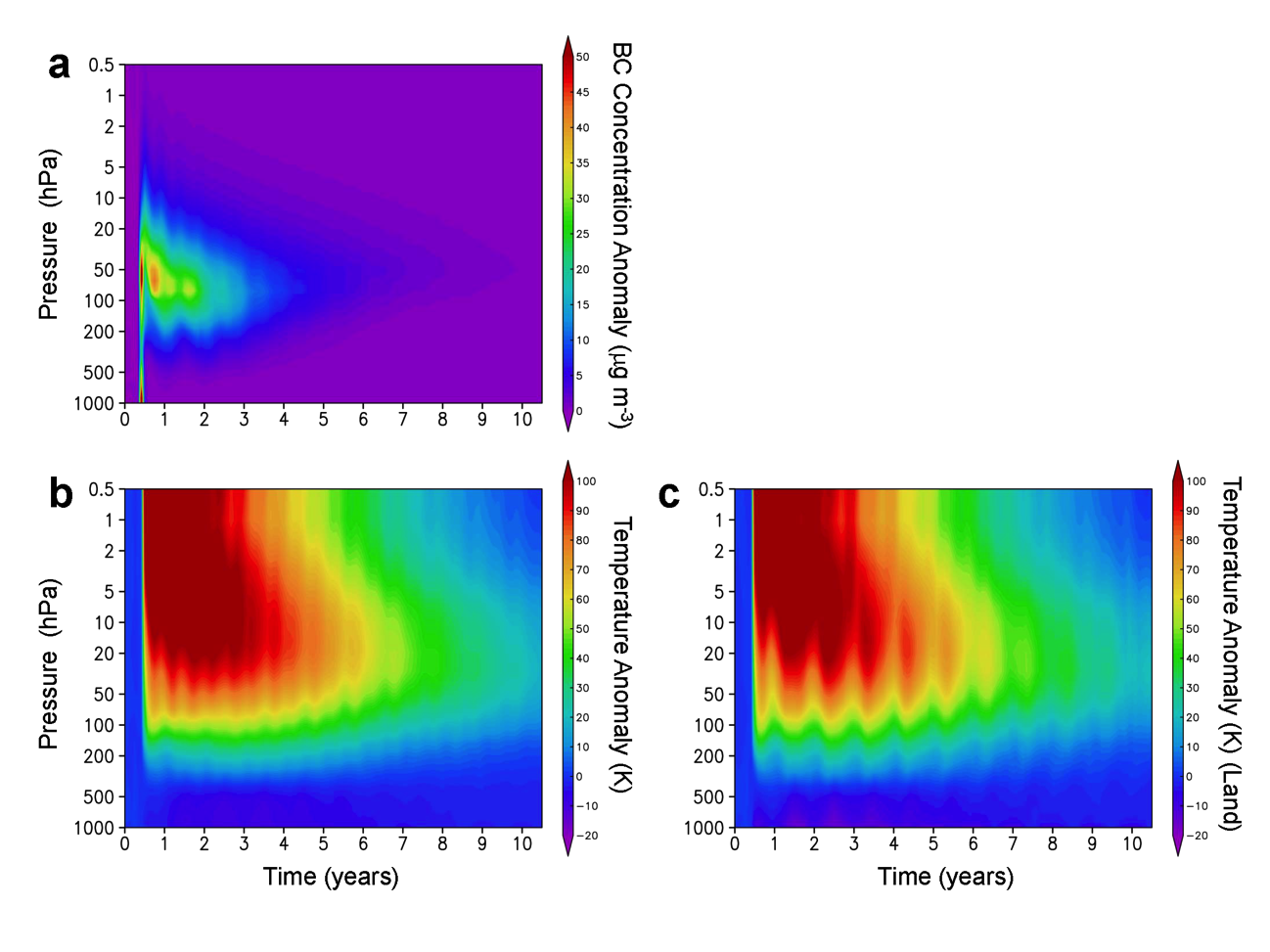


**Supplemental Figure 2. Vertical distributions of climate changes caused by the black carbon (BC) injection.** a–c, Changes in the vertical profiles of global averages of the mass concentration of BC in the atmosphere (a), temperature (b), and temperature over the land (c) for the 1500-Tg BC case, from the surface to 0.5 hPa (approximately 54 km in altitude) calculated by the climate model. Monthly anomalies from the control experiment (no ejection case) are shown.


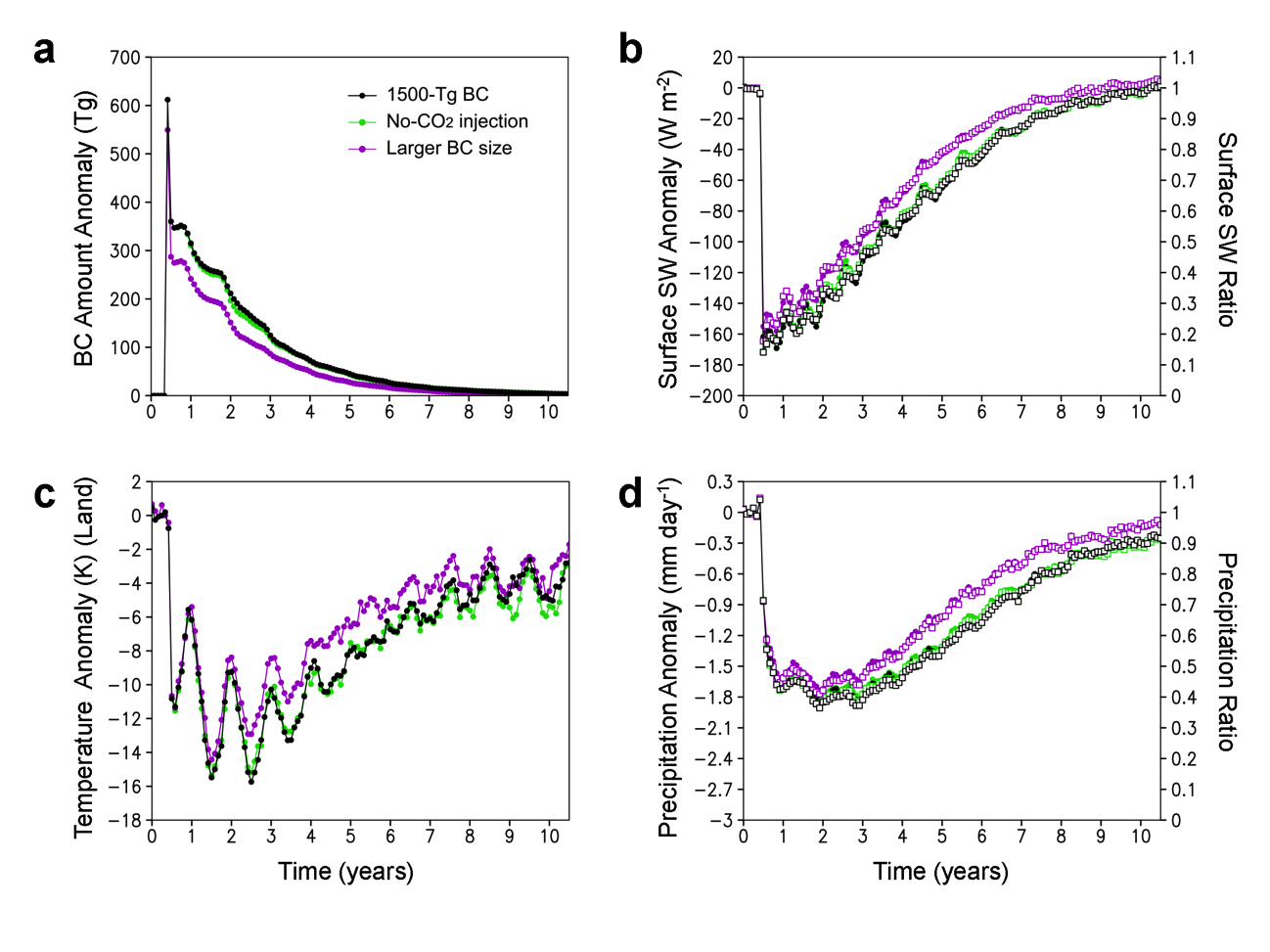


**Supplemental Figure 3. The model sensitivity experiments for the climate changes.** a–d, Changes in the global averages of amount of black carbon (BC) in the atmosphere (a), downward shortwave (SW) radiation at the surface (b), surface air temperature over the land (c), and precipitation (d) for the 1500-Tg BC case (black), the no-CO_2_ injection experiment (green), and the experiment with a larger BC particle size distribution (purple) calculated by the climate model. Monthly anomalies from the control experiment (no ejection case) are shown on the left axis with filled circles (a–d) and the ratios to the control experiment are shown for shortwave radiation and precipitation on the right axis with open squares (b and d).

Supplemental Table 1. Stable carbon isotope ratio, δ^13^C (VPDB) of n-alkanes

| No. | Sample | Height (cm) | Lithology | C16n-Alkane | mV | C16n-Alkane | mV | C16n-Alkane | C29n-Alkane | mV | C29n-Alkane | mV | C29n-Alkane | C31n-Alkane | mV | C31n-Alkane | mV | C31n-Alkane |
| --- | --- | --- | --- | --- | --- | --- | --- | --- | --- | --- | --- | --- | --- | --- | --- | --- | --- | --- |
| 27 | HIST88-91 | 89.5 | Limestone |  |  | -27.72 | 328 | -27.72 |  |  | -31.42 | 94 | -31.42 | -31.24 | 128 |  |  | -31.24 |
| 26 | HIST83-88 | 85.5 | Limestone | -28.84 | 246 | -27.74 | 292 | -27.74 |  |  | -30.55 | 85 | -30.55 | -30.90 | 113 | -31.06 | 84 | -30.90 |
| 25 | HISTIr+2-+3 | 81 | Marl | -31.79 | 315 |  |  | -31.79 |  |  | -31.38 | 315 | -31.38 | -30.48 | 127 | -29.96 | 71 | -30.48 |
| 24 | HISTIr+1-+2 | 80 | Marl | -31.23 | 353 | -30.07 | 496 | -30.07 | -29.44 | 483 | -29.10 | 81 | -29.44 | -29.16 | 134 |  |  | -29.16 |
| 23 | HISTIr0-+1 | 79 | Rust-orange claystone | -32.33 | 305 | -31.04 | 409 | -32.33 |  |  |  |  |  | -29.82 | 102 | -28.97 | 91 | -29.82 |
| 22 | HISTIr-1.5-0 | 77.5 | Marl |  |  |  |  |  |  |  | -29.30 | 70 | -29.30 | -28.44 | 97 | -28.27 | 1580 | -28.27 |
| 21 | HIST70-76 | 73 | Marl | -30.84 | 372 | -30.52 | 558 | -30.52 |  |  | -30.94 | 384 | -30.94 | -30.78 | 293 | -30.49 | 387 | -30.78 |
| 20 | HIST65-70 | 67.5 | Silty v.f. sandstone |  |  | -27.92 | 596 | -27.92 |  |  |  |  |  |  |  |  |  |  |
| 19 | HIST60-65 | 62.5 | Silty v.f. sandstone |  |  | -29.7 | 820 | -29.7 |  |  |  |  |  | -29.17 | 110 |  |  | -29.17 |
| 18 | HIST55-60 | 57.5 | Medium sandstone | -27.17 | 450 | -27.47 | 181 | -27.17 |  |  |  |  |  |  |  |  |  |  |
| 16 | HIST45-50 | 47.5 | Fine sandstone | -27.85 | 59 | -27.45 | 58 | -27.85 |  |  |  |  |  |  |  |  |  |  |
| 15 | HIST40-45 | 42.5 | Fine sandstone | -26.85 | 45 | -26.29 | 44 | -26.85 |  |  |  |  |  |  |  |  |  |  |
| 14 | HIST35-40 | 37.5 | Medium sandstone | -26.93 | 213 | -27.22 | 521 | -27.22 |  |  |  |  |  | -28.42 | 82 |  |  | -28.42 |
| 13 | HIST30-35 | 32.5 | C.-M. sandstone |  |  | -27.48 | 725 | -27.48 |  |  |  |  |  | -28.31 | 183 |  |  | -28.31 |
| 12 | HIST25-30 | 27.5 | Coarse sandstone |  |  |  |  |  |  |  |  |  |  |  |  |  |  |  |
| 11 | HIST20-25 | 22.5 | Very fine sandstone |  |  | -27.54 | 64 | -27.54 |  |  |  |  |  |  |  |  |  |  |
| 10 | HIST15-20 | 17.5 | Very coarse sandstone |  |  |  |  |  |  |  |  |  |  |  |  |  |  |  |
| 9 | HIST10-15 | 12.5 | Very coarse sandstone |  |  |  |  |  |  |  |  |  |  |  |  |  |  |  |
| 8 | HIST0-5 | 2.5 | Granule conglomerate |  |  |  |  |  |  |  |  |  |  |  |  |  |  |  |
| 7 | HIST-2-0 | -1 | Marl | -28.13 | 714 |  |  | -28.13 | -29.48 | 300 |  |  | -29.48 | -29.79 | 746 |  |  | -29.79 |
| 6 | HIST-5--2 | -3.5 | Marl | -28.58 | 1323 | -27.8 | 1570 | -27.8 | -28.84 | 497 |  |  | -28.84 | -28.68 | 198 |  |  | -28.68 |
| 5 | HIST-10--5 | -7.5 | Marl | -26.56 | 473 | -27.24 | 916 | -27.24 | -28.93 | 601 |  |  | -28.93 | -29.63 | 657 |  |  | -29.63 |
| 4 | HIST-15--10 | -12.5 | Marl | -27.23 | 598 | -28.62 | 211 | -27.23 | -28.79 | 127 | -28.22 | 100 | -28.79 | -29.18 | 96 |  |  | -29.18 |

Data in Figure 2 are selected from those data. The selected data are from samples having sharper peak shapes (peak shape rank: green is sharpest, blue is intermediate, greenish yellow is worst). Solid frame indicates a sharper peak shape in the same rank. When there are no difference on the peak shape, we used data from samples having the higher amount shown by mV. The selected data are shown in the white cells.

Supplemental Table 2. Combusted organic molecules from the Beloc stratotype section, Haiti

| Sample | Height (cm) | Sample  wt (g) | OR | TOC (w%) | Cor (ng/g) | Cor (ng/g TOC) | BeP (ng/g) | Bpery (ng/g) | Cor ratio | Cor (ng) | BeP (ng) | Bpery (ng) | CBB (ng) | CBB (ng)/OR | SA (cm^2^) | CBB/OR (ng/cm^2^) |
| --- | --- | --- | --- | --- | --- | --- | --- | --- | --- | --- | --- | --- | --- | --- | --- | --- |
| HIST88～91 | 89.5 | 101.5 | 0.33 | 0.070 | 0.008 | 11.5 | 0.006 | 0.003 | 0.47 | 0.82 | 0.61 | 0.30 | 1.74 | 5.26 | 25 | 0.21 |
| HIST83～88 | 85.5 | 101.6 | 0.2 | 0.064 | 0.039 | 61.6 | 0.025 | 0.019 | 0.47 | 3.96 | 2.54 | 1.93 | 8.43 | 42.16 | 25 | 1.69 |
| HIST Ir.2～3 | 81.0 | 51.1 | 1 | 0.076 | 0.052 | 68.2 | 0.063 | 0.000 | 0.45 | 2.66 | 3.22 | 0.00 | 5.87 | 5.87 | 25 | 0.23 |
| HIST Ir.1～2 | 80.0 | 51.3 | 1 | 0.070 | 0.048 | 68.3 | 0.025 | 0.000 | 0.66 | 2.46 | 1.28 | 0.00 | 3.74 | 3.74 | 25 | 0.15 |
| HIST Ir.0～１ | 79.0 | 50.6 | 1 | 0.061 | 0.115 | 187.2 | 0.036 | 0.007 | 0.73 | 5.82 | 1.82 | 0.35 | 7.99 | 7.99 | 25 | 0.32 |
| HIST Ir.-1.5～0 | 77.5 | 51.0 | 0.66 | 0.061 | 0.085 | 139.3 | 0.439 | 0.000 | 0.16 | 4.33 | 22.37 | 0.00 | 26.70 | 40.45 | 25 | 1.62 |
| HIST72～76 | 74.0 | 101.0 | 0.25 | 0.060 | 0.230 | 385.4 | 0.015 | 0.017 | 0.88 | 23.23 | 1.52 | 1.72 | 26.46 | 105.86 | 25 | 4.23 |
| HIST65～72 | 68.5 | 100.8 | 0.14 | 0.043 | 0.055 | 126.4 | 0.004 | 0.000 | 0.93 | 5.54 | 0.40 | 0.00 | 5.95 | 42.48 | 25 | 1.70 |
| HIST60～65 | 62.5 | 101.1 | 0.2 | 0.054 | 0.547 | 1013.5 | 0.580 | 0.019 | 0.48 | 55.29 | 58.63 | 1.92 | 115.84 | 579.19 | 25 | 23.17 |
| HIST55～60 | 57.5 | 101.3 | 0.2 | 0.036 | 0.040 | 112.4 | 0.005 | 0.000 | 0.89 | 4.05 | 0.51 | 0.00 | 4.56 | 22.79 | 25 | 0.91 |
| HIST50～55 | 52.5 | 100.8 | 0.2 | 0.044 | 0.231 | 527.6 | 0.019 | 0.000 | 0.92 | 23.29 | 1.92 | 0.00 | 25.20 | 126.01 | 25 | 5.04 |
| HIST45～50 | 47.5 | 102.2 | 0.2 | 0.039 | 0.210 | 536.5 | 0.054 | 0.019 | 0.74 | 21.46 | 5.52 | 1.94 | 28.93 | 144.63 | 25 | 5.79 |
| HIST40～45 | 42.5 | 101.0 | 0.2 | 0.038 | 0.110 | 285.8 | 0.024 | 0.000 | 0.82 | 11.11 | 2.42 | 0.00 | 13.54 | 67.68 | 25 | 2.71 |
| HIST35～40 | 37.5 | 101.4 | 0.2 | 0.048 | 0.095 | 199.3 | 0.046 | 0.000 | 0.67 | 9.63 | 4.66 | 0.00 | 14.29 | 71.47 | 25 | 2.86 |
| HIST30～35 | 32.5 | 101.0 | 0.2 | 0.055 | 0.236 | 430.5 | 0.026 | 0.016 | 0.85 | 23.85 | 2.63 | 1.62 | 28.09 | 140.45 | 25 | 5.62 |
| HIST25～30 | 27.5 | 50.7 | 0.2 | 0.053 | 0.081 | 154.5 | 0.015 | 0.000 | 0.84 | 4.11 | 0.76 | 0.00 | 4.87 | 24.36 | 25 | 0.97 |
| HIST20～25 | 22.5 | 80.3 | 0.2 | 0.027 | 0.276 | 1012.5 | 0.112 | 0.023 | 0.67 | 22.17 | 9.00 | 1.85 | 33.02 | 165.10 | 25 | 6.60 |
| HIST15～20 | 17.5 | 100.5 | 0.2 | 0.041 | 0.000 | 0.0 | 0.004 | 0.000 | 0.00 | 0.00 | 0.40 | 0.00 | 0.40 | 2.01 | 25 | 0.08 |
| HIST10～15 | 12.5 | 102.2 | 0.2 | 0.042 | 0.016 | 38.1 | 0.050 | 0.030 | 0.17 | 1.64 | 5.11 | 3.07 | 9.81 | 49.07 | 25 | 1.96 |
| HIST0～5 | 2.5 | 50.6 | 0.2 | 0.043 | 0.000 | 0.0 | 0.015 | 0.000 | 0.00 | 0.00 | 0.76 | 0.00 | 0.76 | 3.80 | 25 | 0.15 |
| HIST-2～0 | -1.0 | 101.9 | 0.5 | 0.059 | 0.046 | 78.3 | 0.078 | 0.000 | 0.37 | 4.69 | 7.95 | 0.00 | 12.63 | 25.26 | 25 | 1.01 |
| HIST-5～-2 | -3.5 | 100.8 | 0.33 | 0.052 | 0.000 | 0.0 | 0.015 | 0.000 | 0.00 | 0.00 | 1.51 | 0.00 | 1.51 | 4.58 | 25 | 0.18 |
| HIST-10～-5 | -7.5 | 100.7 | 0.2 | 0.070 | 0.000 | 0.0 | 0.039 | 0.000 | 0.00 | 0.00 | 3.93 | 0.00 | 3.93 | 19.63 | 25 | 0.79 |
| HIST-15～-10 | -12.5 | 101.9 | 0.2 | 0.083 | 0.030 | 36.6 | 0.138 | 0.088 | 0.12 | 3.06 | 14.06 | 8.97 | 26.09 | 130.46 | 25 | 5.22 |

OR: Occupation rate of samples for the sampling area. Cor: coronene. BeP: benz(e)pyrene. Bpery: benzo(g,h,i)perylene. Cor ratio: Cor/(Cor + BeP + Bpery). CBB: Cor + BeP + Bpery. SA: Sample area.

Supplemental Table 3. Combusted organic molecules from Caravaca, Spain

| Sample | Height (cm) | Sample wt (g) | OR | TOC (w%) | Cor (ng/g) | Cor (ng/g TOC) | BeP (ng/g) | Bpery (ng/g) | Cor ratio | Cor (ng) | BeP (ng) | Bpery (ng) | CBB (ng) | CBB (ng)/OR | SA (cm^2^) | CBB/OR (ng/cm^2^) |
| --- | --- | --- | --- | --- | --- | --- | --- | --- | --- | --- | --- | --- | --- | --- | --- | --- |
| SPCA18～20 | 19 | 50.7 | 0.1 | 0.111 | 0.000 | 0.0 | 0.000 | 0.000 |  | 0.00 | 0.00 | 0.00 | 0.00 | 0.00 | 400 | 0.00 |
| SPCA16～18 | 17 | 50.2 | 0.1 | 0.063 | 0.000 | 0.0 | 0.000 | 0.000 |  | 0.00 | 0.00 | 0.00 | 0.00 | 0.00 | 400 | 0.00 |
| SPCA+14～+16 | 15 | 51.1 | 0.1 | 0.137 | 0.000 | 0.0 | 0.000 | 0.000 |  | 0.00 | 0.00 | 0.00 | 0.00 | 0.00 | 400 | 0.00 |
| SPCA9～10 | 9.5 | 50 | 0.2 | 0.155 | 0.000 | 0.0 | 0.000 | 0.000 |  | 0.00 | 0.00 | 0.00 | 0.00 | 0.00 | 400 | 0.00 |
| SPCA8～9 | 8.5 | 50 | 0.2 | 0.166 | 0.000 | 0.0 | 0.000 | 0.000 |  | 0.00 | 0.00 | 0.00 | 0.00 | 0.00 | 400 | 0.00 |
| SPCA7～8 | 7.5 | 50 | 0.2 | 0.143 | 0.000 | 0.0 | 0.000 | 0.000 |  | 0.00 | 0.00 | 0.00 | 0.00 | 0.00 | 400 | 0.00 |
| SPCA6～7 | 6.5 | 50 | 0.2 | 0.174 | 0.000 | 0.0 | 0.000 | 0.000 |  | 0.00 | 0.00 | 0.00 | 0.00 | 0.00 | 400 | 0.00 |
| SPCA5～6 | 5.5 | 50 | 0.2 | 0.313 | 0.000 | 0.0 | 0.000 | 0.000 |  | 0.00 | 0.00 | 0.00 | 0.00 | 0.00 | 400 | 0.00 |
| SPCA4～5 | 4.5 | 50 | 0.2 | 0.239 | 0.000 | 0.0 | 0.000 | 0.000 |  | 0.00 | 0.00 | 0.00 | 0.00 | 0.00 | 400 | 0.00 |
| SPCA+3～+4 | 3.5 | 51.8 | 0.2 | 0.166 | 0.367 | 219.8 | 0.000 | 0.060 | 0.86 | 19.01 | 0.00 | 3.11 | 22.12 | 110.59 | 400 | 0.28 |
| SPCA+2～+3 | 2.5 | 52.5 | 0.2 | 0.223 | 0.382 | 182.9 | 0.000 | 0.076 | 0.83 | 20.06 | 0.00 | 3.99 | 24.05 | 120.23 | 400 | 0.30 |
| SPCA1～2 | 1.5 | 50 | 0.2 | 0.209 | 0.661 | 581.7 | 0.000 | 0.077 | 0.90 | 33.05 | 0.00 | 3.85 | 36.90 | 184.50 | 400 | 0.46 |
| SPCA+0.5～+1 | 0.75 | 25.9 | 0.4 | 0.254 | 3.832 | 1253.2 | 0.111 | 0.376 | 0.89 | 99.25 | 2.87 | 9.74 | 111.86 | 279.66 | 400 | 0.70 |
| SPCA+0.2～+0.5 | 0.35 | 15.1 | 0.66 | 0.382 | 7.718 | 1579.1 | 0.240 | 0.847 | 0.88 | 116.54 | 3.62 | 12.79 | 132.96 | 201.45 | 400 | 0.50 |
| SPCA0～+0.2 | 0.1 | 15.6 | 1 | 0.253 | 5.773 | 1841.8 | 0.190 | 0.592 | 0.88 | 90.06 | 2.96 | 9.24 | 102.26 | 102.26 | 400 | 0.26 |
| SPCA-0.3～0 | -0.15 | 16.2 | 0.66 | 0.135 | 0.518 | 300.7 | 0.026 | 0.090 | 0.82 | 8.39 | 0.42 | 1.46 | 10.27 | 15.56 | 400 | 0.04 |
| SPCA-0.5～-0.3 | -0.4 | 15.8 | 1 | 0.129 | 0.439 | 248.6 | 0.000 | 0.073 | 0.86 | 6.94 | 0.00 | 1.15 | 8.09 | 8.09 | 400 | 0.02 |
| SPCA-1～-0.5 | -0.75 | 16.8 | 0.4 | 0.113 | 0.598 | 393.1 | 0.035 | 0.082 | 0.84 | 10.05 | 0.59 | 1.37 | 12.01 | 30.01 | 400 | 0.08 |
| SPCA-2～-1 | -1.5 | 50 | 0.2 | 0.101 | 0.049 | 88.8 | 0.000 | 0.004 | 0.93 | 2.45 | 0.00 | 0.20 | 2.65 | 13.25 | 400 | 0.03 |
| SPCA-3～-2 | -2.5 | 50 | 0.2 | 0.091 | 0.152 | 305.6 | 0.047 | 0.064 | 0.58 | 7.60 | 2.35 | 3.20 | 13.15 | 65.75 | 400 | 0.16 |
| SPCA-4～-3 | -3.5 | 52.3 | 0.2 | 0.106 | 0.000 | 0.0 | 0.000 | 0.000 |  | 0.00 | 0.00 | 0.00 | 0.00 | 0.00 | 400 | 0.00 |
| SPCA-5～-4 | -4.5 | 50 | 0.2 | 0.094 | 0.176 | 536.4 | 0.010 | 0.025 | 0.83 | 8.80 | 0.50 | 1.25 | 10.55 | 52.75 | 400 | 0.13 |
| SPCA-7～-5 | -6 | 51.5 | 0.1 | 0.089 | 0.225 | 256.4 | 0.009 | 0.032 | 0.84 | 11.59 | 0.46 | 1.65 | 13.70 | 136.99 | 400 | 0.34 |
| SPCA-9～-7 | -8 | 50 | 0.1 | 0.091 | 0.000 | 0.0 | 0.000 | 0.000 |  | 0.00 | 0.00 | 0.00 | 0.00 | 0.00 | 400 | 0.00 |
| SPCA-11～-9 | -10 | 50 | 0.07 | 0.084 | 0.000 | 0.0 | 0.000 | 0.000 |  | 0.00 | 0.00 | 0.00 | 0.00 | 0.00 | 400 | 0.00 |
| SPCA-13～-11 | -12 | 52.1 | 0.1 | 0.106 | 0.000 | 0.0 | 0.000 | 0.000 |  | 0.00 | 0.00 | 0.00 | 0.00 | 0.00 | 400 | 0.00 |
| SPCA-15～-13 | -14 | 50.2 | 0.1 | 0.090 | 0.000 | 0.0 | 0.000 | 0.000 |  | 0.00 | 0.00 | 0.00 | 0.00 | 0.00 | 400 | 0.00 |
| SPCA-17～-15 | -16 | 51.8 | 0.02 | 0.099 | 0.000 | 0.0 | 0.000 | 0.000 |  | 0.00 | 0.00 | 0.00 | 0.00 | 0.00 | 400 | 0.00 |

OR: Occupation rate of samples for the sampling area. Cor: coronene. BeP: benz(e)pyrene. Bpery: benzo(g,h,i)perylene. Cor ratio: Cor/(Cor + BeP + Bpery). CBB: Cor + BeP + Bpery. SA: Sample area.

**Supplemental Table 4. Monthly averaged ocean surface air temperatures (°C) and seawater temperatures (°C) in the 0-Tg BC scenario and the temperatures (°C)**

| Amount of BC |  |  | 0 Tg BC | |  |  |  | 500 Tg BC* | | |  |  | 1500 Tg BC* | | |  |  | 2600 Tg BC* | | |  |
| --- | --- | --- | --- | --- | --- | --- | --- | --- | --- | --- | --- | --- | --- | --- | --- | --- | --- | --- | --- | --- | --- |
| Latitude / Water depth (m) | A2^+^ | 2^#^ | 50^#^ | 100^#^ | 200^#^ | 400^#^ | 2 | 50 | 100 | 200 | 400 | 2 | 50 | 100 | 200 | 400 | 2 | 50 | 100 | 200 | 400 |
| 30°N–45°N January | 10–15 | 13–18 | 14–19 | 14–19 | 13–18 | 11–16 | 4 | 3 | 3 | 2 | 1 | 5 | 5 | 5 | 4 | 2 | 6 | 6 | 6 | 5 | 3 |
| 30°N–45°N July | 15–20 | 17–22 | 10–15 | 10–15 | 8–13 | 6–11 | 7 | 3 | 3 | 2 | 2 | 10 | 5 | 4 | 4 | 2 | 11 | 6 | 6 | 5 | 3 |
| 15°N–30°N January | 15–22 | 17–24 | 17–24 | 17–24 | 13–20 | 8–15 | 4 | 4 | 3 | 2 | 1 | 7 | 7 | 6 | 4 | 1 | 9 | 9 | 8 | 6 | 2 |
| 15°N–30°N July | 20–25 | 21–26 | 18–23 | 16–21 | 13–18 | 8–13 | 5 | 4 | 3 | 2 | 1 | 9 | 7 | 6 | 4 | 2 | 11 | 9 | 8 | 6 | 2 |
| 0–15°N January | 22–27 | 23–28 | 23–28 | 20–25 | 14–19 | 8–13 | 3 | 2 | 2 | 1 | 1 | 7 | 7 | 4 | 3 | 1 | 10 | 9 | 6 | 3 | 1 |
| 0–15°N July | 25–27 | 26–28 | 25–27 | 22–24 | 16–18 | 10–17 | 3 | 2 | 2 | 1 | 1 | 8 | 7 | 4 | 3 | 1 | 10 | 9 | 7 | 3 | 2 |
| 0–15°S January | 25–27 | 27–29 | 25–27 | 22–24 | 15–17 | 9–11 | 3 | 2 | 2 | 1 | 1 | 8 | 7 | 4 | 2 | 1 | 11 | 9 | 6 | 3 | 1 |
| 0–15°S July | 23–25 | 24–26 | 24–26 | 21–23 | 14–16 | 9–11 | 3 | 2 | 2 | 1 | 1 | 7 | 7 | 4 | 2 | 1 | 10 | 9 | 6 | 3 | 1 |
| 15°S–30°S January | 20–25 | 21–26 | 18–23 | 16–21 | 13–18 | 7–12 | 5 | 4 | 3 | 2 | 0 | 9 | 7 | 6 | 3 | 1 | 11 | 9 | 7 | 5 | 1 |
| 15°S–30°S July | 17–20 | 18–21 | 18–21 | 17–20 | 14–17 | 8–11 | 4 | 4 | 3 | 2 | 0 | 7 | 7 | 6 | 3 | 1 | 8 | 8 | 7 | 5 | 1 |
| 30°S–45°S January | 15–20 | 15–20 | 11–16 | 11–16 | 9–14 | 8–13 | 5 | 4 | 3 | 3 | 2 | 8 | 6 | 5 | 4 | 3 | 10 | 7 | 6 | 5 | 3 |
| 30°S–45°S July | 13–15 | 15–17 | 14–16 | 14–16 | 14–16 | 10–12 | 4 | 4 | 3 | 3 | 2 | 6 | 6 | 6 | 4 | 3 | 7 | 7 | 7 | 5 | 3 |

^*^The maximum decreasing seawater temperatures at each water depth in each BC ejection case of the climate model calculations of this study (Fig. 6).

^+^Latest Cretaceous monthly mean surface air temperature (2 m height) over ocean according to the averaged values from the BESTGUESS and BARESOIL simulations of Upchurch et al.40. ^#^Estimates by subtracting the difference between surface air temperature over ocean and sea water temperature at each water depth of the monthly climatological temperature of the control experiment (30-year mean in the pre-industrial condition) from the A2 temperature.
